# The Cantabria Cohort, a protocol for a population-based cohort in northern Spain

**DOI:** 10.1186/s12889-023-17318-8

**Published:** 2023-12-05

**Authors:** Marta Alonso-Peña, Trinidad Dierssen, Maria José Marin, Jessica Alonso-Molero, Inés Gómez-Acebo, Inés Santiuste, Jeffrey V. Lazarus, Pascual Sanchez-Juan, Galo Peralta, Javier Crespo, Marcos Lopez-Hoyos, Cantabria Cohort Collaborators, Ana Peleteiro-Vigil, Bernardo Alio Lavin Gomez, Olga Alvaro Melero, Maria Teresa Arias-Loste, Ana Batlle, Joaquin Cabezas, Jorge Calvo Montes, Joaquín Cayon de las Cuevas, Laura Conde, Lara Diego Gonzalez, Carmen Fariñas, Sara Fernandez Luis, Maria Fernandez Ortiz, Santiago Garcia Blanco, Gema Garcia Lopez, Maite Garcia Unzueta, Jose Carlos Garrido Gomez, Raquel Gonzalez, Paula Iruzubieta, Jesus Martin Lazaro, Lucia Martin Ruiz, Nerea Martinez Magunacelaya, Raul Martinez Santiago, Juan Manuel Medina, Maria Josefa Muruzabal Siges, Ana Padilla, Ana Peleteiro, Luis Reyes-González, David Ruiz, Alvaro Santos-Laso, Maria Elena Sanz Piña, David Sordo, Sergio Solorzano, Rafael Tejido, Reinhard Wallman, María Wunsch

**Affiliations:** 1 Valdecilla Research Institute (IDIVAL), Santander, 39011 Spain; 2 Faculty of Medicine, University of Cantabria, Santander, 39011 Spain; 3 Barcelona Institute for Global Health (ISGlobal), Hospital Clínic, University of Barcelona, Barcelona, Spain; 4 CUNY Graduate School of Public Health and Health Policy (CUNY SPH), New York, NY USA; 5 CIBERNED, Network Center for Biomedical Research in Neurodegenerative Diseases, National Institute of Health Carlos III, 28220 Madrid, Spain; 6 Alzheimer’s Centre Reina Sofia-CIEN Foundation-ISCIII, 28031 Madrid, Spain; 7 Marques de Valdecilla University Hospital, Santander, 39008 Spain

**Keywords:** Population-based cohort, Lifestyle, Socio-economic factors, Biobank, Big data, Precision medicine, Longitudinal study, Spain

## Abstract

Cantabria Cohort stems from a research and action initiative lead by researchers from Valdecilla Research Institute (IDIVAL), Marqués de Valdecilla University Hospital and University of Cantabria, supported by the regional Goverment. Its aim is to identify and follow up a cohort that would provide information to improve the understanding of the etiology and prognosis of different acute and chronic diseases. The Cantabria Cohort will recruit between 40,000–50,000 residents aged 40–69 years at baseline, representing 10–20% of the target population. Currently, more than 30,000 volunteers have been enrolled. All participants will be invited for a re-assessment every three years, while the overall duration is planned for twenty years. The repeated collection of biomaterials combined with broad information from participant questionnaires, medical examinations, actual health system records and other secondary public data sources is a major strength of its design, which will make it possible to address biological pathways of disease development, identify new factors involved in health and disease, design new strategies for disease prevention, and advance precision medicine. It is conceived to allow access to a large number of researchers worldwide to boost collaboration and medical research.

**Supplementary Information:**

The online version contains supplementary material available at 10.1186/s12889-023-17318-8.

## Background

Recent reports have shown that non-communicable diseases, especially cardiovascular and metabolic diseases, were the main cause of morbi-mortality in Spain in 2019 [1]. Therefore, risks factors related to lifestyle, behavior and environment are placing a heavy burden on the Spanish population’s health [1]. At the same time, incidence of these diseases and an aging population threaten the sustainability of the Spanish universal health system. That stated, preventive, predictive and personalized medicine are urgently needed; to achieve such an aim, extensive data and knowledge will be fundamental.

Long-term follow-up cohort studies have played a crucial role in understanding modifiable factors associated with the development of chronic disease. In the early 1950s, initial results from the Framingham Heart Study were published [2, 3], beginning a new era in epidemiology [4]. Large prospective cohort studies have established themselves as the most appropriate epidemiological design for research in the field of multimorbidity in real-life conditions [5]. Over the last decades, the number of large prospective cohorts have increased [6–10].

The Framingham Heart study selected its study population from a well-defined geographic area, the city of Framingham (Massachusetts, USA). This same strategy was carried out in Europe. One of the most remarkable examples was that of the European Prospective Investigation into Cancer and Nutrition (EPIC), which was an initiative of the International Agency for Research on Cancer (IARC) [6]. More recently, national cohort studies have been developed [7–10].

In Spain, several efforts have been undertaken to establish prospective, population-based cohorts [11–19]. However, to date, long-term, population-based and multipurpose studies covering a specific territory have not been attemped. Conversely, a national cohort study has just been launched [20]. Health system management in Spain takes place at the regional (autonomous community) level, which in practice results in 17 autonomous health systems within Spain [1].

**Fig. 1:**
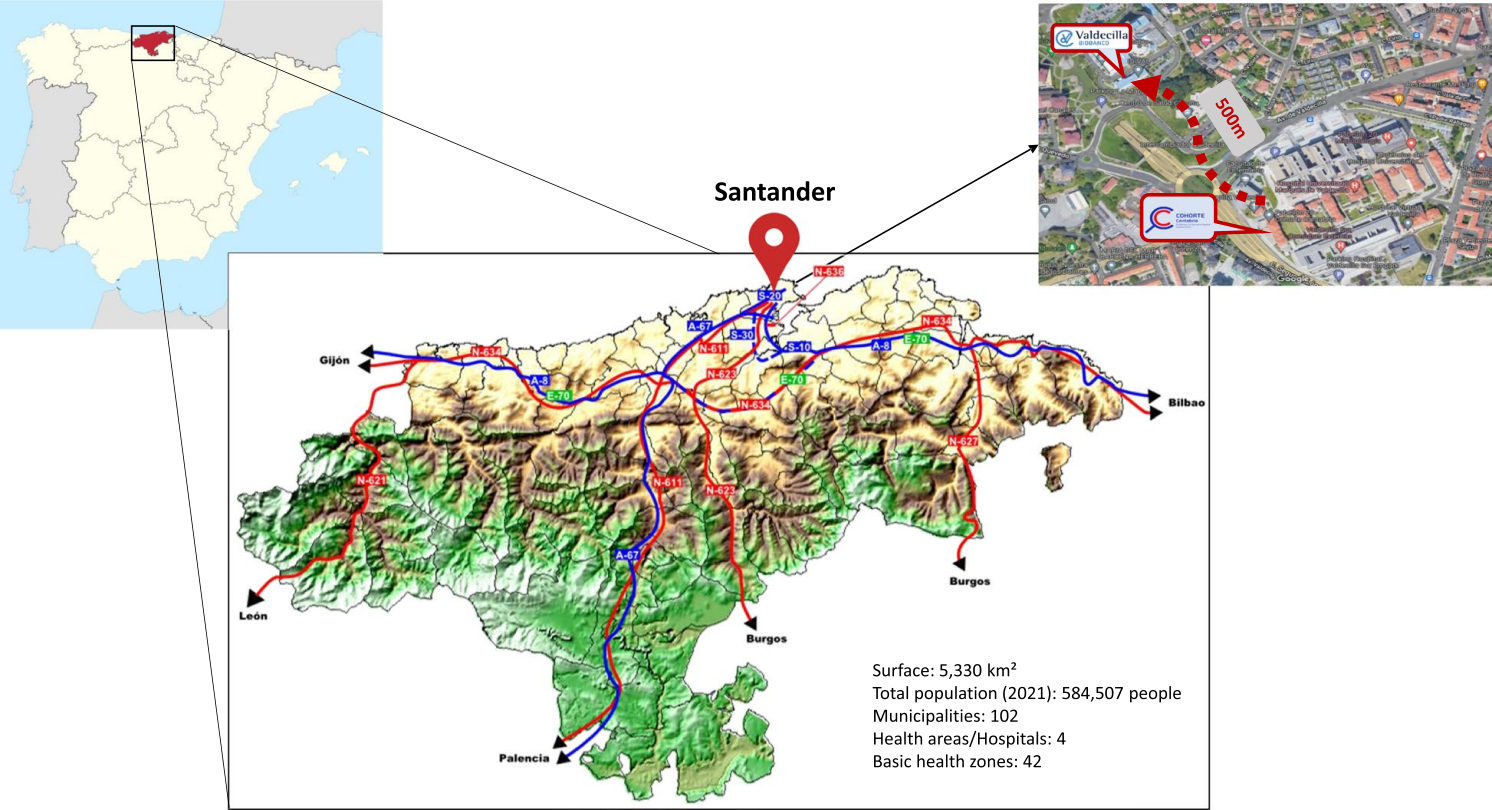
Location of study centers and main transport connections in the region [21]. Santander is the region’s capital. *Map source: Wikimedia Commons*

In this context, the Cantabria Cohort was launched in late 2020 and it includes residents in Cantabria, an autonomous community located in northern Spain. Cantabria covers an area of 5,330 km^2^, hosting a total population of 584,507 inhabitants, 45% living in the region’s capital [21] (Fig. 1). Moreover, the Cantabrian Public Health System is organized into 42 health areas for primary care. It has four hospitals; the Hospital Universitario Marqués de Valdecilla (HUMV) is the tertiary and referenced hospital in the region. This center helps implement large-scale studies, reducing the necessity of coordination among different institutions and procedures. Further, due to the widespread COVID-19 vaccination campaign in 2021, a huge effort to integrate multiple public databases and update contact information of the population has facilitated the start of the project. Cantabria Cohort stems from a research and action initiative to improve the regional health system and advance the health-related Sustainable Development Goals [22]. The present paper describes the design and implementation of the Cantabria Cohort and the processes related to the biological sample collection and data acquisition. It also provides an overview of the governing board, quality assurance, and legal and ethical aspects, as well as future research opportunities and cooperation.

## Study objectives

This project’s main objective is to identify and follow up a cohort that would provide baseline information on lifestyles, socio-economic aspects, and morbidity of the Cantabrian population. This information would be related to health events registered during the follow-up to improve the understanding of the etiology and prognosis of different acute and chronic diseases. The repeated collection of biomaterials combined with broad information from participant questionnaires, medical examinations, actual health system records and other public data sources is a major strength of its design. The study is planned and built from a multidisciplinary research and action perspective. It is conceived to allow access to a large number of researchers worldwide to boost collaboration and medical research. Overall, five general objectives are pursued by the Cantabria Cohort. Within their general scope, more specific research questions and projects will be included.

### Integration and use of real-world data to improve health research

One of the main objectives is the development of systems to use official and public secondary data sources for scientific research. This implies the integration, validation and analysis of dispersed data, as well as the legal and administrative governance necessary to assure the safety and ethical use of the sources. In this regard, real-world evidence is an emerging domain in health research and several European projects have been launched to create collaborative networks in which clinical data can be shared and integrated to define common data models, standardize vocabulary and delineate government structures, such as the European Health Data & Evidence Network (EHDEN) [23]. Cantabria Health Service and Valdecilla Research Institute (IDIVAL) joined EHDEN. Our project has taken advantage of the first steps taken to create a dataset using the Observational Medical Outcomes Partnership (OMOP) Common Data Model [24] and boost its transformation into a longitudinal database for implementation in research. Official data sources other than health records will also be included in the project.

### Identifying lifestyle risk factors and their involvement in chronic diseases

The first set of aims is to increase the understanding of the role of lifestyle factors (e.g., smoking, alcohol consumption, physical activity, dietary patterns, body composition, occupation and environmental conditions) in developing major forms of chronic disease, with a special emphasis on obesity, metabolic syndrome and its related conditions. Of note, 60.4% of the Cantabria population is considered to have a sedentary lifestyle [25] and incidence of such conditions is increasing worldwide [26]. By integrating different sources of data, as described below, we aim to gain insights into the natural history and causal physiopathological pathways. In the Cantabria region, up to 27% of the population older than 15 years old has hypercholesterolemia, 22.6% suffers from hypertension, 19% low-back pain, 15.2% mental illness, 7.9% has diabetes, 5.6% arthrosis and 1.7% suffers from chronic obstructive pulmonary disease [27]. We also aim to document the impact of these risk factors in terms of epidemiology and public health strategies.

### Evaluation of geographic and socio-economic disparities in health and healthcare

Another objective of the Cantabria Cohort is to increase awareness about the causes of social and regional disparities in health. According to the National Health System Annual Report 2020–2021, the positive perception of health in the population aged 15 and over is clearly lower among people with a basic level of education and below, especially among women [25]. Besides, the AROPE (At risk of poverty or social exclusion) rate in Cantabria is 19.4% [25]. Socio-economic position and psychosocial factors will be evaluated as health determinants via calculation of HOUSES index [28] from primary questionnaires and Spanish Cadastre and ecological secondary (Atlas of Urban Vulnerability) socio-economic sources of data [29, 30]. Finally, information will be collected on the use of health services, medical interventions and medications.

### Assessing biomarkers for the early detection of diseases and disease risks

Determining biomarkers has become one of the main objectives of biomedical research. Technological advances, especially in “omic” sciences, have revolutionized research on disease biomarkers and even the identification of health markers. However, applying such “omics” requires large collections of biological samples to be consistent and avoid statistic bias [31]. Therefore, one of the main objectives of Cantabria Cohort is to collaborate with other international partners in the investigation and development of omics studies to identify new health and disease biomarkers, increase biological replicates and introduce diversity into the genetic and environmental backgrounds of the populations being analyzed.

### Surveillance of viral hepatitis and HIV in Cantabria

The World Health Organization (WHO) set the ambitious goals of eliminating viral hepatitis B and C as a public health threat by 2030 and reducing the number of people newly infected with HIV [26]. To achieve these goals, the scale-up of direct-acting antiviral therapies is a necessary cornerstone. It cannot, however, be implemented without micro-elimination programmes that facilitate identification of undiagnosed cases. Once diagnosed, viral hepatitis and HIV can be treated, efficiently reducing the risk of transmission [32, 33]. Furthermore, HCV, HBV and HIV are spread by the same mechanisms, so it is common that individuals infected with one of these viruses are also infected by the others [34]. According to the last reports of the Spanish Directorate General of Public Health, in collaboration with the Cantabrian Autonomous System of Epidemiological Surveillance, the notification of new VIH cases has been reducing from 53 total cases in 2009 to 14 in 2020 [35], while the incidence of hepatitis B is the highest in the country (2.23 cases per 100,000 inhabitants in 2020) [36] and that of hepatitis C is also among the highest in Spain (2.75 cases per 100,000 inhabitants in 2020) [37]. Thus, the Cantabria Cohort also aims to reduce HCV, HBV and HIV incidence in Cantabria and enhance its treatment by serological testing in all participants.

## Study design and methods

For the development and execution of these study protocol, we have follow the recommendations of SPIRIT 2013 guideline [38].

### Study population and recruitment

**Fig. 2:**
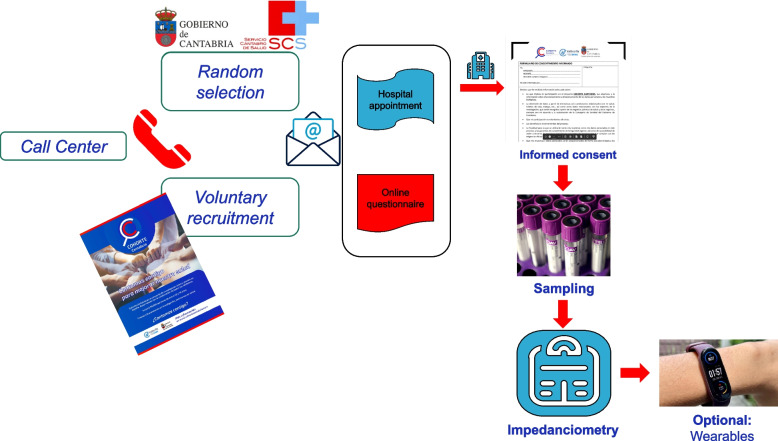
Selection process, patient recruitment and collection of information for the study

The Cantabria Cohort will recruit 40,000–50,000 residents aged 40–69 years at baseline. An information campaign in various general media was launched in 2021. Thereafter, study participants were recruited through any of the following: 1) voluntary registration on the study website (www.cohortecantabria.com) or direct telephone contact and 2) random selection (stratified by sex and age) using the Cantabrian Public Health System population database. Afterwards, participants are contacted by telephone; they are informed about all the phases of the study, their rights of withdrawal, the possible disadvantages of participating in the study, and other fundamental characteristics. People who agree to participate are sent the Informed Consent together with the full Participant Information Sheet and an appointment at the study center, HUMV (Fig. 2). Recruitment and calls are carried out by trained personnel and will continue until the sample size is reached or follow-up is initiated, which is planned for April 2024.

No financial compensation for participation is planned, but participants receive, if requested, a report of their test results (i.e., blood test results and anthropometric data). Further, the medical team reviews all discordant analytical results and health records to decide whether any clinical finding should be communicated to the study participants or need further medical attention. Recruitment started on 20 April 2021 and it is still ongoing. The expected response rate in the global randomly selected population was around 50%, which was confirmed during recruitment (response rate in the whole randomly selected population is currently 43.6%). At the moment of publication, more than 30,000 individuals have already been included in the study, of whom 57.8% were randomly selected. Due to potential bias in recruitment, type of recruitment (voluntary/random) has been recorded for each individual in the study. Once recruitment is closed, deep analysis will be undertaken and published to compare the demographics of the voluntary group and the random group against each other (See preliminary data on Supplementary Table 2), and against the Cantabrian population. Moreover, in order to evaluate the reproducibility of our findings, we will perform sensitivity analysis that excludes voluntarily registered participants. Data to be released for future research will always include the variable "type of participation" so that researchers can include it in multivariate models to control for its potential confounding effect.

### Baseline examination and data collection

Data collection starts with the telephone call wherein current contact information is updated, if necessary. Once identification, contact information and appointment have been confirmed, a self-administrated questionnaire is delivered as a link to REDCap platform via email [39]. It can also be given on paper in a format ready for digital reading data extraction in the case of non-Internet users. Digital questionnaires must be completed before the appointment while paper questionnaires must be returned by post or in person.

Medical examinations last 10–15 min and include blood extraction for biobanking and basic analytics and measurements of anthropometric characteristics [40]. Finally, a random subset of participants is invited to carry a wearable activity wristband.

All participants will be re-invited for follow-up examinations (re-assessment) every three years after their baseline recruitment, including approximately the same examinations.

#### Laboratory analysis and biological samples

Biological material from all patients will be treated in accordance with Law 14/2007 on Biomedical Research [41]. Blood samples will be used to measure conventional analytical parameters as well as serological markers of viral infection (Table 1, Supplementary Table 1). If the participant expressly authorizes the donation of samples to the Valdecilla Biobank, two more tubes of blood are withdrawn. In addition, authorization is requested for integration into the Valdecilla Biobank of the surplus tissue samples from therapeutic or diagnostic surgical procedures available at Cantabrian hospitals’ pathology departments. The samples kept in the custody of the Biobank will be governed by the provisions of Royal Decree 1716/2011, of November 18 [42].

**Table 1:** Data obtained at baseline examination from laboratory, impedanciometry and questionnaires

Source	Category	Data/Instrument
**Laboratory**	Biochemistry	Serum glucose, urea, creatinine, sodium, potassium, uric acid, ALT, AST, GGT, alkaline phosphatase, triglycerides, HDL, LDL and total cholesterol, albumin, calcium, C-reactive protein
	Hormones	TSH, T4
	Hepatology	APRI, FIB4
	Haematology	Leukocytes, neutrophils, lymphocytes, monocytes, eosinophils, basophils, erythroblasts, red blood cells, hemoglobin, hematocrit, mean corpuscular hemoglobin, erythrocyte distribution width, mean corpuscular volume, mean corpuscular hemoglobin concentration, platelets, mean platelet volume
	Microbiology	Hepatitis B, Hepatitis C, HIV
**Impedanciometry**	Anthropometric measurements	Height, weight, body mass index, waist circumference
	Body composition	Fat mass, visceral adipose tissue, lean mass, skeletal muscle mass, total body water, extracellular water
	Energy	Energy expenditure, energy expenditure at rest, stored energy
	Raw impedanciometry data	Bioimpedance vector, impedance, reactance, resistance, phase angles
**Questionnaire**	Socioeconomic	Marital status, level of education, employment status, annual income, housing characteristics, place of residence
	Demographic	Age, sex, ethnicity, phototype
	Quality of life	Spanish version of the SF-36 Health Survey [43]
	Diet	13-item Mediterranean diet adherence screener and 9-item scale of adherence to a low-fat diet [44]
	Physical activity	Spanish version of the physical activity questionnaire [45]
	Sleep habits	The items employed to get this information come from GCAT Genomes for life project [15]
	Tobacco use/exposure	GCAT Genomes for life project [15]
	Alcohol consumption	GCAT Genomes for life project [15]
	Family history of disease	Progenitors, siblings and descendants. These items were got from GCAT Genomes for life [15]
	Memory	Cognitive Complaints Questionnaire [40]

*ALT* Alanine transaminase, *AST* Aspartate transaminase, *GGT* Gamma-glutamyltransferase, *HDL* High-density lipoprotein, *LDL* Low-density lipoprotein, *TSH* Thyroid-stimulating hormone, *T4* Thyroxine, *APRI* AST-to-Platelet Ratio Index, *FIB4* Fibrosis index based on four factors, *HIV* Human immunodeficiency virus

#### Study questionnaire

This questionnaire includes the level of education attained, gross income, quality of life, diet, physical activity, family history, work activity and other habits, anthropometric data, tobacco and alcohol consumption, housing characteristics, etc., measured by externally validated surveys (Table 1, Supplementary Table 1). The questionnaire is structured in six independent modules to ease its completion and requires approximately one hour.

#### Body composition

Bioelectric impedance analysis (BIA) has emerged as a validated method for evaluating body composition [40] and detecting malnutrition [46, 47] (Table 1, Supplementary Table 1). Seca mBCA 515/514 and 274 digital stadiometers (Hans E. Rüth, Barcelona, Spain) are used in this context to obtain an immediate overview of the distribution of muscles, fat and water in the body by 8-point bioelectrical impedance analysis of 19 different frequencies and seven body segments.

#### Wearable activity wristband

Participants may be offered an electronic bracelet (Xiaomi MI Smart Band 5) that will allow us to obtain information on: 1) physical activity; 2) sleep; and 3) heart rate. Specifically, a measurement of steps and the type of physical activity is performed every minute while heart rate, every 10 min for 21 days. Afterward, the device is connected to the researchers’ smartphones as previously described [48]. Until now, around 10% of study participants have accepted to wear the electronic bracelet and provide information on daily physical activity.

#### Data from public health records

Cantabria Health Service and IDIVAL joined EHDEN in 2020 [49]. Currently, the IDIVAL database in EHDEN is a cross-sectional dataset representing citizens who receive public health assistance from the Cantabrian Health Service between 2016 and 2020. The information provided by the region’s primary care is related to annotations, diagnoses (converted from ICPC2 to SNOMED), clinical variables and vaccines. From the hospital setting, appointments, tests (SNOMED), diagnoses (converted to SNOMED from IDC10), variables and specific information on Hospital Pharmacy have been included. Laboratory results (LOINC) and electronic prescription information from both settings are included. Data transformation and validation were performed as previoculy described [50]. The Cantabria Cohort will take advantage of the inclusion of its target population in IDIVAL dataset. Thus, all information regarding public health assistance is directly linked to Cantabria Cohort Database. Furthermore, procedures have been developed to use national healthcare databases to allow for the identification and validation of diseases throughout follow-up.

Non-users of public health assistance from the Cantabrian Health Service will be identified and asked to provide updates on their health records every three years. Until now, only 2.4% of recruited participants are non-users.

Data on main diagnoses and comorbidities obtained from health databases will be validated annually from the 10% of the randomly chosen sample. The validation will be performed on a selection of the most prevalent diagnoses in the cohort. The reference criterion for validation will be a review of clinical history by two trained physicians to evaluate the sample independently and in masked form. Internal validity parameters will be calculated: sensitivity and specificity with their 95% CI and external validity (predictive values). In addition, inter-rater concordance will be performed using the kappa index.

#### Other data sources

In addition to data from public health services, there is a multitude of documentary sources from different public and governmental administrations with information that can help to understand the living conditions of the participants and their relevance in health and disease. For this reason, Cantabria Cohort works with administrations to facilitate the secondary use of data for research. Among them, the following draw attention:

- National Death Index (IND) from Spanish Ministry of Health: The deaths in the cohort will be updated annually based on the IND.
- Spanish Cadastre from the Spanish Ministry of Finance and Public Administration: based on the cadastral reference, information about real state and housing will be imported (square meters of the state, cadastral value, location, etc.).
- The Urban Vulnerability Atlas developed by the Ministry of Transport, Mobility and Urban Agenda: sociodemographic, socio-economic, housing and subjective perception vulnerabilities.
- Social Security System from the Spanish Ministry of Inclusion, Social Security and Migration: it will allow the incorporation of participants' labor information.
- Data collected by the Spanish National Institute of Statistics.

### Time line and statistical power considerations

**Fig. 3:**
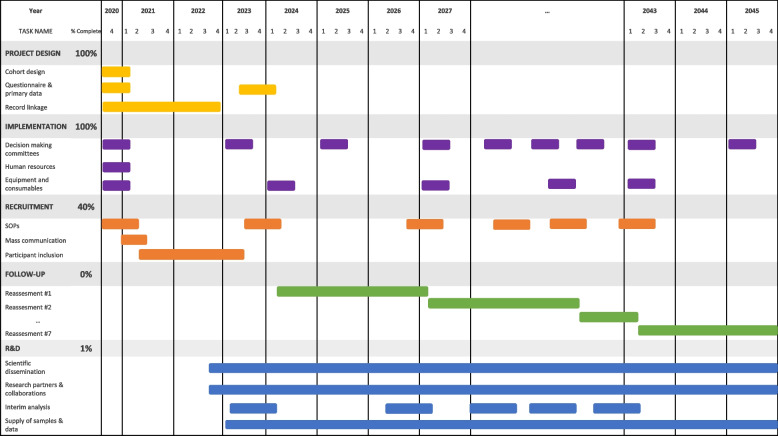
Cantabria Cohort project execution and future development – timeline. SOPs: standard operating procedure. R&D: Research and development

**Fig. 4:**
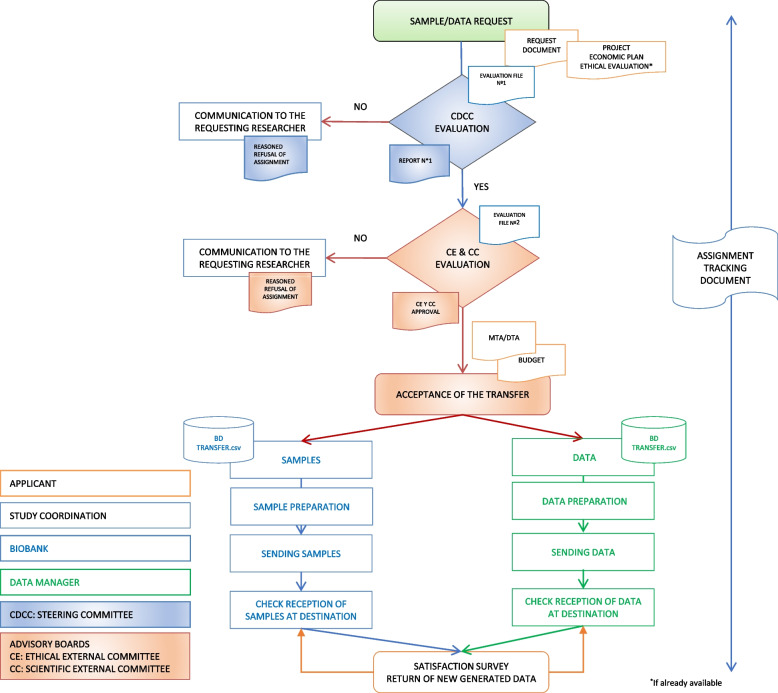
Statistical power per hazard ratio at 5 (**A**) and 20 (**B**) years of follow-up

The projected timeframe for the Cantabria Cohort covers 20 years (Fig. 3) starting 2021. For the calculation of statistical power, we have considered two follow-up time points: at 5 and 20 years, after which we expect to have retained 75% (35,000) and 10% (5,000) of the initial sample, respectively (Fig. 4). Estimating the sample power to detect weak associations (hazard ratios less than 1.2) allows us to evaluate the study's capacity to identify associations in the worst-case scenario, which needs the largest sample size. Furthermore, hazard ratio enables the control of the confounding effect of differential follow-up times, therefore it has been proposed employing Hazard Ratio estimation to calculate the necessary sample size to achieve optimal statistical power at the conclusion of the study [51, 52]. Thus, with 35,000 individuals, the minimum hazard ratio values that can be detected with an 80% power are 1.1, 1.12, 1.14 and 1.2 for effect ratio values of 10%, 7.5%, 5% and 2.5%, respectively; whereas, with 5,000 individuals, the minimum hazard ratio values that can be detected with an 80% power are 1.29, 1.34, 1.43 and 1.65 for effect ratio values of 10%, 7.5%, 5% and 2.5%, respectively.

## Study organization

### Central data management

Data will be collected trough web-based, REDCAP standardized data entry forms, while others sources of data (analytical parameters, impedance, webereables, paper-based questionnaires) will be transformed from their original database into csv files to be easily handled in different statistical software packages. These sources of data will be validated and integrated into a SQL database to be readily available upon request of data collection services, ensuring continuous back-ups of the full datasets. Statistical analysis will depend on the project and specific research aims. However, as a general rule, the data will be presented differently based on the type of variable. For qualitative variables, absolute frequencies and percentages will be used. For quantitative variables, measures of central tendency like mean and median, as well as measures of dispersion like standard deviation, interquartile range, minimum and maximum values will be used to provide indicators of the distribution's shape, such as asymmetry indices and kurtosis. In the case of ordinal variables, the description used will depend on the number of categories. Regardless of the variable type, a column will be added to the data presentation table to indicate the number of patients with available data.

All data will be kept in secure servers from the regional health service. Prospective record linkage will be performed by the Digital Transformation team from the Regional Ministry of Health of the Government of Cantabria, a trusted third party separate from the main study database. The trusted third party maintains all linkages to external secondary data sources.

### Collection and storage of biological samples

Samples collected for future research are registered, processed and stored at the Valdecilla Biobank, an active member of the Spanish Platform ISCIII Biobanks and Biomodels. For this purpose, two tubes of blood are drawn from all participants who agree to donate a sample to the biobank:

- 10 ml Serum separator clot activator tube
- 10 ml K2EDTA tube.

Samples are processed according to the SOPs of the Spanish Biobanks Network to obtain serum, plasma and buffy coat (BC) or DNA. Handling and storage of the samples is performed in the same location where obtained, ensuring the quality and stability of the biological samples by following the ISO 9001:2015 standard. Plasma and serum samples are aliquoted into six 2D cryotubes each and stored at -80ºC within two hours of collection. BC samples remain at -40 °C until DNA extraction. A magnetic bead nucleic acid extraction system (Chemagic 360, PerkinElmer Inc) is used to isolate DNA from the BC. A biorepository management software is used to efficiently and securely manage biological samples, guaranteeing the complete traceability of sample information and all their associated data (Noraybanks, Noray Bioinformatics, S.L.U.)

### Ethics and data confidentiality

All participants in the study receive written information about the project and the Informed Consent document at home and are also informed verbally about it (by telephone call to make an appointment). If they agree to participate, they sign the Informed Consent form on the day of the appointment at the study center where nurses and other healthcare personnel can resolve any other questions or doubts. The Informed Consent has been designed for collection and use of participant data and biological specimens in ancillary studies.

The study protocol (ID 2021.057) was approved by the Ethics Committee for Drug Research of Cantabria (CEIm) on 26 February 2021. The study respects the ethical principles of research with biological samples, the 1975 Declaration of Helsinki and Spanish Law 14/2007, of July 3, 2007, on Biomedical Research. Specifically, restrictions to sample and data used given by participants through informed consent will always be revised and respected before any sample or data transfer. Furthermore, the study have been registered in ClinicalTrials.gov as “The Cantabria Cohort, a Protocol for a Population-based Cohort in Northern Spain” (NCT05852678), being the protocol version 3.0 on December 2, 2022.

The processing, communication and transfer of personal data of all participants will comply with the provisions of the applicable regulations: Regulation (EU) 2016/679 of the European Parliament and of the Council of 27 April 2016 on Data Protection (RGPD) and the Organic Law 3/2018, of 5 December, on the Protection of Personal Data and guarantee of digital rights [53]. Thus, samples and data accessible to the public will always be transmitted in a pseudo-anonymized form and specific SOPs and security mechanisms will be implemented to avoid re-identification. Specifically, to guarantee the privacy and confidentiality of the information obtained, two sets of data were generated, stored in encrypted form:

1. A main database, only accessible by the study's data manager, with the identification data of the participants together with their identification codes in the different sources of data and related tables.
2. For every data transfer, an anonymized database in which a numerical code is assigned to each participant will be generated. This dataset gathers all the information collected in this study and requested by researchers.

In compliance of the Organic Law 3/2018 on the Protection of Personal Data and guarantee of digital rights and Spanish Law 14/2007 on Biomedical Research, any important protocol modifications and information regarding third parties accesing to the data will be communicated broadly through the project webpage, www.cohortecantabria.com, and participants will be specifically informed on a quarterly basis through a newsletter.

### Sample and data transfer

**Fig. 5:**
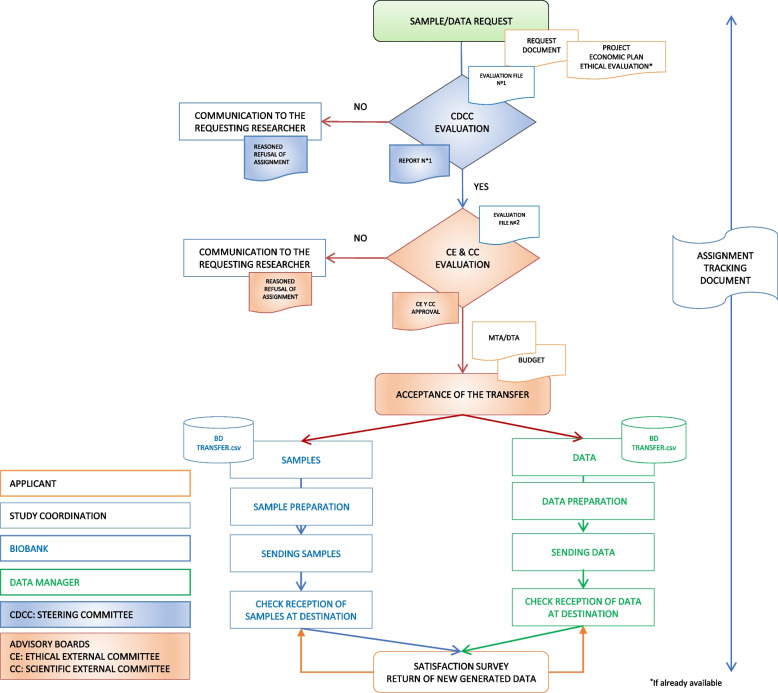
Flowchart for Cantabria Cohort’s data and sample transfer to research groups and institutions

The Cantabria Cohort has a firm commitment to participants and researchers to establish, develop and promote health research and the advancement of medicine and strategies to improve the well-being of the Cantabrian society. Samples and data from the Cantabria Cohort may be used for biomedical research projects by public biomedical research centers, universities, or private for-profit and not-for-profit institutions. Due to the limited nature of the samples and the strategic nature of the associated information, which is their main added value, sample and data transfer will be controlled by the Steering Committee (Fig. 5) and will be based on the following principles:

- The Cantabria Cohort will publish on its website its policy and procedures for access to samples and data for use in research.
- The Cantabria Cohort will maintain full control over access to and use of project data and samples in accordance with its commitment to public use.
- The Cantabria Cohort Steering Committee will ensure compliance with the collection access policy to ensure that the resource is used efficiently and for public benefit.

Once the Cantabria Cohort Steering Committee authorizes the transfer of samples and/or data, the request will be sent for evaluation to the Ethical External Committee and the External Scientific Committee (Fig. 5).

Regarding authorship eligibility, the number of authors will depend on the specific requirements of each journal, with the maximum number of authors allowed as a limit. In order to facilitate maximum impact for the greatest number of authors participating in each of the ancilliary studies of Cantabria Cohort, all the papers derived directly from the project will include a first, third and last authors proposed by the principal investigator of the study and a senior co-author proposed by the Steering Committee. Furthermore, the publication must express the acknowledgement to the Cantabria Cohort and IDIVAL.

### Funding, governance and management

**Fig. 6:**
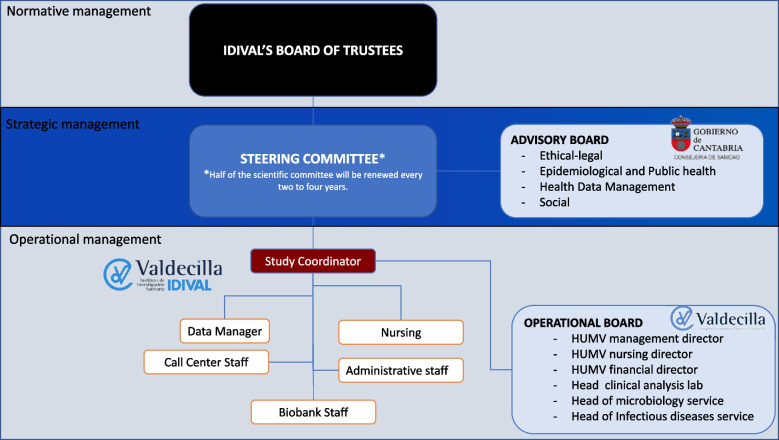
Scheme of governance and management structures of the Cantabria Cohort

This project has the institutional support of all the health research organizations in our community. It is led by IDIVAL, with all participating researchers being the heads of the research unit of their respective groups (Javier Crespo, Trinidad Dierssen, Marcos López Hoyos and Pascual Sánchez). In addition, Marcos López Hoyos is the scientific director of IDIVAL; Galo Peralta, the managing director of IDIVAL and María José Marín, the Scientific Director of the Valdecilla Biobank. The Cantabria Cohort has public endorsements from the Regional Minister of Health of the Government of Cantabria, the manager of the Cantabrian Health Service, the rector of the University of Cantabria, the director of IBBTEC and the managing director of HUMV (Fig. 6). This study protocol was not independently peer reviewed as part of the funding process. The initial budget, which covered the first 8 months of recruitment, amounted to an investment of 1.5 million euros. It is estimated that annual costs will increase to 1.6–1.8 million euros.

## Conclusion

The Cantabria Cohort has been designed as a multi-purpose prospective cohort and research tool to investigate any acute and chronic illnesses, especially those associated with lifestyle, nutrition, exercise and aging. This project sets extraordinary groundwork for scientific cooperation and networking among epidemiologists and other health scientists, improving our regional health system and advancing the national and global achievements of health-related United Nations’ Sustainable Development Goals.

### Supplementary Information

**Additional file 1.**

**Additional file 2.**

## Data Availability

The datasets used and/or analysed during the current study available on reasonable request following standard procedures as described in the manuscript, “Sample and Data Transfer” section. Contact information and rules for accession will be updated on www.cohortecantabria.com. For inquiries related to this article, please contact corresponding author.
