# Supplementary material for: The Cantabria Cohort, a protocol for a population-based cohort in northern Spain
Source: BMC Public Health. 2023 Dec 5;23:2429. doi: 10.1186/s12889-023-17318-8 (PMC10698930; doi:10.1186/s12889-023-17318-8)
Supplement: Supplementary file 2 — Additional file 2. [file 12889_2023_17318_MOESM2_ESM.docx]

**Supplementary Table 2**: Sociodemographic characteristics of Cantabria Cohort participants according to recruitment method (random selection or voluntary recruitment) until 02/10/2023.

| **Variable** | **Random selection**  **(n = 17,803)** | **Voluntary recruitment**  **(n = 12,973)** | **p*** |
| --- | --- | --- | --- |
| Gender (female, %) | 54.1 | 66.7 | 5.2×10^-109^ |
| Age (years, mean±SD) | 55.7±8.6 | 53.7±8.4 | 4.7×10^-89^ |
| Educational level (%) |  |  | 0.0 |
| No education  Primary school  Secondary school, 1st cycle  Vocational education  Secondary school, 2nd cycle  Higher vocational education  University studies | 0.8  13.4  15.4  13.8  12.4  17.6  26.1 | 0.3  6.2  9.3  11.0  11.9  17.4  43.8 |  |
| Gross annual income (%) |  |  | 0.0 |
| < 18,000€  18,000 – 30,999€  31,000 – 51,999€  52,000 – 100,000€  > 100,000€ | 17.6  31.0  24.3  10.7  1.4 | 13.3  27.7  30.5  18.2  2.1 |  |

*p-values were calculated by t-test for age and chi-squared test for the remaining categorical variables. SD: Standard deviation.
